# Adverse Childhood Experiences (ACEs) Screening in Primary Care Settings for Adults: A Systematic Review

**DOI:** 10.1007/s40653-025-00691-4

**Published:** 2025-02-12

**Authors:** Betül Küçükardalı-Cansever, Angela Lamson, Natalia Sira, Stephanie Ann Bridgland, Sheena Eagan, Erin Roberts

**Affiliations:** 1 Department of Human Development and Family Science, College of Health and Human Performance, East Carolina University, Mailstop 505 Greenville, Greenville, NC 27858 USA; 2 Department of Psychology, East Carolina University, Greenville, NC USA; 3 Department of Bioethics and Interdisciplinary Studies, East Carolina University, Greenville, NC USA

**Keywords:** Adverse childhood experiences, Primary care, Systematic review, ACEs screening, Biopsychosocial health

## Abstract

Substantial evidence links ACE scores with adult health complications, emphasizing the significance of enhanced detection, interpretation, and management of ACEs in primary care settings for adults. This systematic review aims to explore existing ACE screening and post-screening practices for adult patients in primary care settings worldwide via a thorough systematic review, including a search of PubMed, PsycINFO, and CINAHL databases. Out of an initial 883 potential articles from two independent reviewers, 53 articles met the inclusion and exclusion criteria. Most studies were conducted in the US (71.7%). Globally, 83% of the ACE screening studies indicated that ACES were screened specifically for research purposes, while the remaining 17% covered quality improvement, trauma-informed care interventions, and routine screenings in healthcare settings. This review delves into various intricacies of ACEs screening, the professional roles involved in the screening process, the location where the screening was conducted, prior training on ACEs for the screening implementers, and subsequent actions like interventions, referrals, and resource allocation. Despite growing awareness of the long-term effects of ACEs, a gap exists between ACEs research and related clinical practices in primary care. Additionally, there is no consensus on how to utilize ACEs screening in primary care and which ACE scores necessitate indicated interventions. Implications focus on the collaborative health use of ACEs screenings in primary care.

**Supplementary Information:**

The online version contains supplementary material available at 10.1007/s40653-025-00691-4.

## Introduction

Adverse Childhood Experiences (ACEs) range from abuse and neglect to growing up with a caregiver who was incarcerated and are widely recognized for their profound impact on long-term health outcomes. Madigan et al. (2023) conducted a comprehensive meta-analysis, which included data from 546,458 adults across 208 studies. The results revealed that 60% of people globally are estimated to have experienced at least one form of childhood adversity. Specifically, it is estimated that 22% experienced one ACE, 13% experienced two ACEs, 9% experienced three ACEs, and 16% experienced four or more ACEs, whereby the higher the score, ultimately the higher the risk for mental and physical health concerns in adulthood. In the United States, approximately 61% of individuals are estimated to have had at least one negative childhood experience, and 16% have experienced at least four ACE adversities (Merrick et al., 2019). In response to the high prevalence and significant impact of ACEs, health policy and practice organizations have started advising physicians to screen for ACEs. Primary care is particularly well-suited to address ACE-related concerns due to its preventive and interventive nature and its accessibility to people from diverse backgrounds (Glowa et al., 2016). However, ACE screening involves very personal and sensitive questions that may negatively impact individuals, especially those with high ACE scores, unless accompanied by trauma-informed care and appropriate interventions or referrals (Austin et al., 2024; Baca & Salsbury, 2023; Racine et al., 2019). Despite the recognition of these needs, the ACE screening practices in primary care settings remain under-explored.

## Adverse Childhood Experiences

Adverse Childhood Experiences (ACEs) refer to a range of potentially traumatic events occurring between the ages of 0 and 18, which can have a direct negative impact on a child’s development (Felitti et al., 1998). In their seminal study, Felitti et al. (1998) categorized these adverse events into ten items across three categories: *abuse* (emotional, physical, and sexual), *neglect* (emotional and physical), and *household dysfunction* (maternal violence, substance abuse within the household, household mental illness, parental separation, and an incarcerated household member). The ACE score, which quantifies the range of adverse childhood experiences, is calculated by counting the number of different types of adversity an individual was exposed to, yielding a score ranging from 0 to 10. A score of 0 indicates no exposure to any ACE categories, while a score of 10 reflects exposure to all ACE categories. This score serves as a measure of cumulative childhood stress and its potential biopsychosocial repercussions (Ports et al., 2020).

Flaherty et al. (2009), one of many research teams who has recognized the long-range ramifications of ACEs, found that adverse childhood events are common even in the earliest childhood years and have the capability of contributing to poor health and somatization. Dube et al. (2006) recognized a “dose-response” relationship between ACE scores and early alcohol use, establishing a strong link between ACEs and alcohol initiation by age 14. Similarly, Felitti (2009) argued that coping mechanisms such as overeating, smoking, drug use, and promiscuity mediate the effects of childhood adversities on adult behavioral issues and chronic health conditions. More specifically, Gilbert et al. (2015) found that ACE exposure increased the chances of myocardial infarction, asthma, fair/poor health, frequent mental distress, and disability compared to individuals with no ACE exposure. Additionally, those with four to nine ACEs had greater probabilities of coronary heart disease, stroke, and diabetes. Numerous studies have consistently shown that higher ACE scores correlate with an increased risk of adverse health outcomes, including physical and mental health issues, engagement in risky health behaviors, diminished life satisfaction, and premature death (Brown et al., 2009; Felitti et al., 1998; Merrick et al., 2017; Metzler et al., 2017).

While ACEs affect many people, structural and social circumstances that influence how individuals interact with their environments place certain groups at higher risk (Andersen & Blosnich, 2013; Merrick et al., 2018a, b). Several studies have identified disparities in ACE exposure based on race and ethnicity (Kenney & Singh, 2016; Sacks & Murphey, 2018). For example, the prevalence of ACEs is higher among underrepresented racial groups compared to non-Hispanic whites (Ports et al., 2020). Additionally, gay/lesbian and bisexual individuals have a 66% and 58% higher incidence rate ratio (IRR) for ACEs than heterosexual individuals, respectively (Andersen & Blosnich, 2013). Importantly, ACEs are not caused by demographic categories such as race, class, or gender. However, minoritized groups often face increased social and economic challenges and limited access to healthcare, which contribute to pathways leading to biopsychosocial health disparities (Ports et al., 2020).

### Primary Care for ACEs Inquiry

Primary care is characterized by the delivery of coordinated, easily accessible health services by clinicians responsible for meeting a significant portion of patients’ individual healthcare needs, forging enduring relationships with patients, and working within the context of the patient’s family and community (Institute of Medicine [U.S.] Committee on the Future of Primary Care, 1996). Primary care providers, such as internists, family doctors, pediatricians, or non-physician practitioners (e.g., family nurse practitioners and physician assistants), play a crucial role in this context (Shi, 2012), and access to their services is linked to positive health outcomes (Starfield et al., 2005). The high frequency of ACEs, along with evidence showing their connection to widespread, expensive, and debilitating health issues (U.S. Centers for Disease Control and Prevention, 2024), provides a strong rationale for ACE screening programs and interventions (Rariden et al., 2020) in health contexts. Proponents suggest that routine ACE inquiries in healthcare settings could help identify individuals at higher risk for negative health outcomes and potentially inform care and treatment choices (Glowa et al., 2016). Primary care is argued to be well-suited for this role due to its preventive and interventive nature and its accessibility to diverse populations. By incorporating ACE screening into routine practice, primary care providers may be better positioned to address the needs of both pediatric and adult patients. However, while there is a strong advocacy for ACE screening, it has not been universally adopted or formally recommended as a preventive care strategy by professional societies or the U.S. Preventive Services Task Force. Currently, less than 30% of primary care physicians report routinely implementing ACE screening (Maunder et al., 2020; Weinreb et al., 2010).

Furthermore, Watson (2019) argues that ACEs meet the evidence-based criteria for screening: they are prevalent, can be detected, and the conditions they are associated with have research-based early treatments. Despite this perspective, it is noted that most physicians do not routinely screen for ACEs (Watson, 2019). This “knowledge-to-action gap” indicates that study data have not yet been integrated into standard professional practice (Watson, 2019; Kalmakis et al., 2017; Weinreb et al., 2010). A recent scoping review (Mishra et al., 2023) provided further insights into the feasibility and benefits of ACE screening in primary care settings. The review found that ACE screening can be integrated into routine healthcare without significantly disrupting workflows, with most screening conversations lasting between 3 and 10 min. Providers generally supported ACE screening, recognizing its potential benefits but also expressed a need for more training and resources. Patients were receptive to ACE screening, appreciating the opportunity to discuss their trauma histories with empathetic clinicians. Mishra et al’s review highlighted the importance of proper training and resources to handle ACE screenings effectively, including trauma-informed care practices and mental health resources. Implementation challenges such as time constraints, lack of training, and concerns about re-traumatization were noted. Despite these barriers, the review concluded that ACE screening could serve as a valuable tool for initiating discussions about trauma and guiding care and treatment choices (Mishra et al., 2023).

### Trauma-Informed Care

Studies indicate that in primary care settings, promoting ACE screening for adults requires a shift in perspective that goes beyond merely implementing the screening itself; it necessitates a broader approach, which is trauma-informed care (TIC; Substance Abuse and Mental Health Services Administration, 2014). TIC is a comprehensive framework designed to embed system-level interventions within healthcare organizations. It integrates the 4Rs: realizing that trauma exists, recognizing the signs and symptoms, responding by creating trauma-informed policies, and resisting re-traumatization. This approach is grounded in six key principles: safety, trustworthiness, and transparency; peer support; collaboration and mutuality; empowerment, voice, and choice; and cultural, historical, and gender issues (Substance Abuse and Mental Health Services Administration, 2014).

Implementing TIC in primary care settings is crucial due to the high prevalence of trauma among patients and its significant impact on health outcomes. Screening for trauma and childhood adversities is an integral aspect of TIC, yet it requires comprehensive preparation. Physicians and their offices must be equipped to provide necessary aftercare, such as treatment, referrals, or emotional support, before initiating any screening (Leasy et al., 2019). Additionally, educating all clinic staff on how to work with patients who screen positive for trauma is essential (Raja et al., 2015), as is incorporating trauma-informed language in both documentation and patient interactions (Leasy et al., 2019). TIC emphasizes building trusting relationships with patients, offering informed choices, and ensuring safety within clinical spaces. This approach is particularly important for individuals who have faced power abuses, such as childhood abuse, political persecution, or systemic racism (Gopal et al., 2023).

Addressing trauma in primary care is essential because of its causal links to mental and physical health changes. Encouraging healthcare professionals to uncover past traumas can enhance their understanding of the relationship between a patient’s trauma and their current health issues. This practice can also improve clinician-patient relationships and facilitate patient healing by acknowledging their trauma. However, implementing screening questionnaires that reveal intimate and traumatic experiences must be managed carefully to avoid re-traumatizing patients (Leasy et al., 2019). Additionally, there is concern that both healthcare providers and patients may be emotionally unprepared to handle the potential traumas and negative emotions that may arise during screening (Albaek et al., 2018; Watson, 2019).

### Purpose

The objective of this study is to systematically review existing ACE screening practices for adult patients in primary care settings worldwide. Different cultures and institutions may have distinct patient populations with unique needs and varying resources or approaches for ACE screening. While substantial literature exists on ACEs, there has yet to be a comprehensive synthesis of screening practices, availability of TIC interventions, and referrals following positive ACE screenings. Primary care, pediatrics, social work, rehabilitation, nursing, and medical family therapy are just some fields that benefit from this contribution to science. These disciplines have dedicated themselves to disease prevention, with a focus on health outcomes, health disparities, health management, health education, and health economics. Therefore, it is essential to determine how the findings of the ground-breaking ACEs study have reverberated in the primary care field, which is frequently utilized in healthcare procurement.

With this systematic review, we aim to investigate and synthesize the process of screening adult patients (18 years of age and older) for ACEs only in primary care, including the purposes for which these scores were used and the clinical services implemented in response to ACE screening. The research questions to be answered are:

- RQ1: What are the primary objectives of conducting ACE screenings in adult patients within primary care settings? (e.g., as part of a TIC quality improvement project, to introduce an intervention, to collect ACE information for research purposes)
- RQ2: What are the conditions and procedures for conducting ACE screening for adults? (e.g., is a private environment provided, are appropriate training provided to researchers/clinic staff)
- RQ3: How do researchers or healthcare personnel utilize the ACE scores obtained from participants? (e.g., to explain the relationship between scores and health problems to participants, to include participants in intervention groups, to identify correlations with specific risk factors or diagnoses)
- RQ4: What specific clinical services are provided following the identification of high ACE scores in adult patients in primary care settings? (e.g., certain trauma-informed practices, providing resources, referrals, or interventions)

## Methods

### Search Strategy

This systematic review was conducted according to the Preferred Reporting Items for Systematic Reviews and Meta-Analysis (PRISMA) guidelines (Page et al., 2021). With the assistance of a medical librarian, we conducted a comprehensive literature search in three databases (the Cumulative Index of Nursing and Allied Health Literature, PubMed, and PsycINFO) using the following keywords: ‘adverse childhood experiences’ OR ‘adverse childhood experience’ OR ACE OR ‘childhood adversity’ AND ‘Primary care’ OR ‘primary healthcare’ OR ‘internal medicine’ OR ‘family practice’ OR ‘family medicine’ OR ‘ob/gyn’ OR ‘Ob-gyn’ OR Obstetrics OR Gynecology OR Gynaecology OR ‘clinic visit’ OR ‘clinic visits’ OR ‘clinical settings’ OR ‘clinical setting’ OR ‘general practice’ OR ‘general practitioners’ OR ‘general practitioner’ OR ‘family doctor’ OR ‘family doctors’ OR ‘primary care provider’ OR ‘primary care providers’ OR ‘nurse practitioner’ OR ‘nurse practitioners’ OR APRNS OR NPs OR ‘nurse-midwives’ OR ‘nurse-midwife’ OR ‘physician assistant’ OR ‘physician assistants.’ This review included publications that met inclusion and exclusion criteria up through July 5, 2024. Since the first large-scale research on adverse childhood experiences (ACEs) did not appear until the late 1990s (Felitti et al., 1998), it was unlikely that any peer-reviewed papers satisfying the qualifying criteria had been published before this time. There was no restriction placed on the search with respect to the location or language of the studies considered.

### Eligibility Criteria

Articles were included for the full-text review if the: (1) original ACE study screening (Centers for Disease Control and Prevention, 2021) or ACE International Questionnaire (ACE-IQ) (WHO, 2020) (i.e., a questionnaire asked about the history of abuse and neglect, parental substance use or mental illness, parental incarceration, domestic violence, or parental divorce) was used within primary healthcare services (e.g., internal medicine or family medicine or general practice or gynecology or Ob/GYN); (2) study sample included participants aged 18 years and older; (3) ACEs screening was completed either by the patient or the health care professional either on-site or off-site; (4) study was an original study with qualitative/quantitative/mixed methods; (5) study was published in a peer review journal or was an unpublished part of a finished dissertation. We did not restrict study location, language, or sample size. Studies that focused primarily on ACE screening but also included social determinants of health (i.e., economic and social factors) were included if they met all the inclusion criteria. Furthermore, studies that evaluated ACEs screening when it was completed as part of a trauma-informed care approach were also included if they met all the inclusion criteria. Articles were excluded if: (1) their sample population was children or adolescents (< 18 years); (2) they were not data-based (e.g., books, theoretical papers, reviews); (3) they did not use the original ACEs questionnaire (Centers for Disease Control and Prevention, 2021); (4) they examined the impact of only one ACE (e.g., divorce); (5) their study setting was only in a mental health clinic; or (6) it was a secondary data study with no available information about how ACEs screening was conducted.

## Data Extraction and Synthesis

**Fig. 1:**
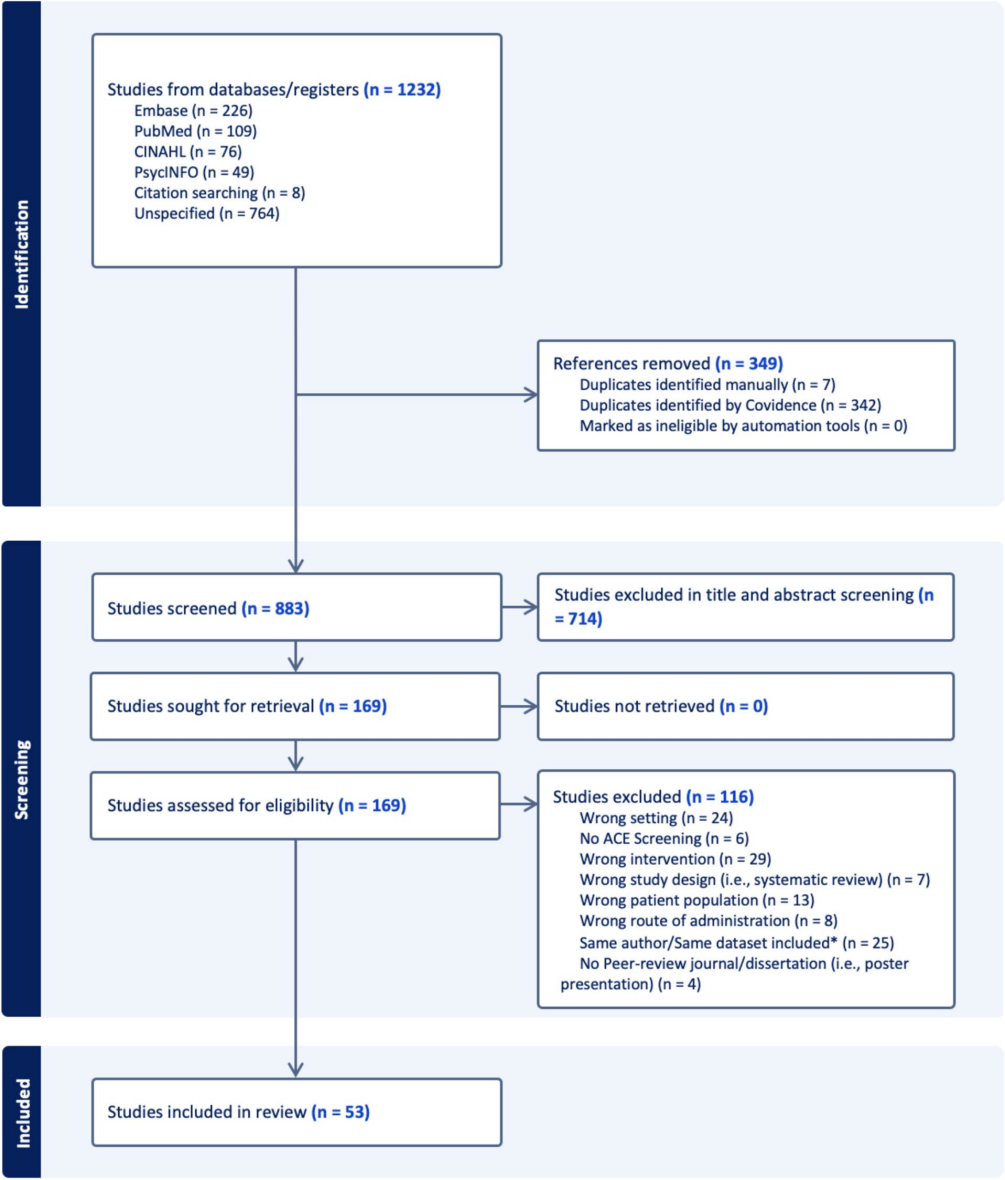
PRISMA flow diagram of study selection. *Some researchers published multiple papers using the same dataset to answer different research questions. To avoid duplicating participants, only the first published article or the one with the largest sample size from each dataset was included, as long as it provided relevant information on ACE screening procedures. This ensures that data collected once from participants is not counted multiple times

Initial searches covered the interval from Felitti et al.‘s (1998) original study to July 5, 2024. Throughout the selection process, two reviewers independently evaluated (in differing portions) the retrieved articles to determine their eligibility for inclusion. Discrepancies between the two reviewers on which articles should move to the full-text review stage were resolved through discussion. If a consensus could not be reached, a third reviewer was consulted to make the final decision. (i.e., the second author). The combined database search yielded 1,232 studies; after removing duplicates, 883 studies remained (See Fig. 1 for a detailed overview). In the second step, we removed irrelevant articles based on the review of titles and abstracts. Cohen’s κ determined moderate agreement between reviewers at the title and abstract review stage, κ = 0.583. Following a review of the title and abstract, 169 potentially relevant articles were identified and subjected to a second round of full-text evaluation by two reviewers. Cohen’s κ determined substantial agreement between reviewers at the full-text review stage, κ = 0.763. Discrepancies during the full-text review stage were resolved similarly, with a third reviewer consulted if necessary.

Of the 169 articles subjected to full-text evaluation, 53 met the eligibility criteria for finding answers to our research questions (see Fig. 1). We extracted detailed information from the selected studies, including the country/state, sample sizes, and study classification. We also gathered data on sample demographics, such as race, ethnicity, and gender percentages, and the number of ACEs items measured. Additionally, we documented procedures on how ACEs were screened, including screening location, method of administration, and the profession of the administrator. Narrative descriptions of how researchers utilized the ACE scores were included, for instance, informing patients, providing resources, or using the data solely for analysis. We examined the clinical services provided upon ACE screening within the research protocol, such as providing resources, arranging referrals, and conducting interventions. Furthermore, we noted the availability of translated ACEs for non-native participants, the inclusion of ACE-related staff/researcher training into the study design, and the inclusion of fidelity measures. This comprehensive approach ensured a thorough understanding of the impact and implementation practices of ACE screenings in primary care settings. The studies included in this review are listed in the Supplementary file.

Based on the premise strongly advocated by the TIC approach that screening for positive ACE scores without an intervention plan may be problematic, we aimed to understand the researchers’ motivations for ACE screening in primary care by carefully examining the purpose and methodology sections of the included studies. During our detailed review of the full-text articles, we sought to identify the underlying rationale for ACE screening, even if it was not explicitly stated in the study. If a study explicitly mentioned including ACE screening for quality improvement purposes, routine screening practices, or testing the effectiveness of interventions for individuals with positive ACE scores, we categorized the study accordingly. For example, if a study reported testing the impact of trauma-informed care interventions, it was categorized as a “trauma-informed care trial.” Similarly, if a study noted that ACE screening was already part of the institution’s routine clinical practice, we classified it under “routine ACE screening.” In cases where the studies did not provide any context or rationale regarding the purpose of ACE screening in their setting, we classified them under the “research purposes only” category. These studies often used ACE screening solely as one of the measures for data collection and analysis. Ultimately, four categories emerged from this analysis: (1) Research purposes only, (2) Quality improvement testing, (3) Trauma-informed care trials, and (4) Routine ACE screening.

During our detailed review of full-text articles, we also identified how ACE scores were utilized, even if not explicitly stated. By analyzing study objectives, methodologies, and findings, we documented notes on their application. Seven categories emerged from this analysis: (1) correlations with health risk behaviors, (2) correlations with disease diagnoses, (3) identifying healthcare access, nonattendance, literacy, referrals, protective factors, and/or social determinants of health, (4) identifying ACE patterns in specific groups, (5) discussions between medical providers and patients, (6) eligibility criteria for interventions, and (7) screening-related preferences. These categories highlight the diverse applications of ACE scores. Studies using ACE scores in statistical models aligned with categories (1) and (2), while those exploring patterns in specific groups matched category (4). Clinical discussions and intervention planning fell under categories (5) and (6). Where studies did not explicitly describe ACE score use, we inferred their utilization based on context, ensuring a comprehensive understanding of their role in primary care research and practice.

## Results

### Overview of Included Studies

This systematic review includes a total of 53 studies, of which 7 are doctoral dissertations. Studies were conducted in the United States (*n* = 38), Canada (*n* = 8), the United Kingdom (*n* = 1), the Federation of Bosnia and Herzegovina (*n* = 1), Ireland (*n* = 1), Iraq (*n* = 1), Australia (*n* = 1), Mexico (*n* = 1), and Lebanon (*n* = 1). Studies were published between 2001 and 2024.

**Fig. 2:**
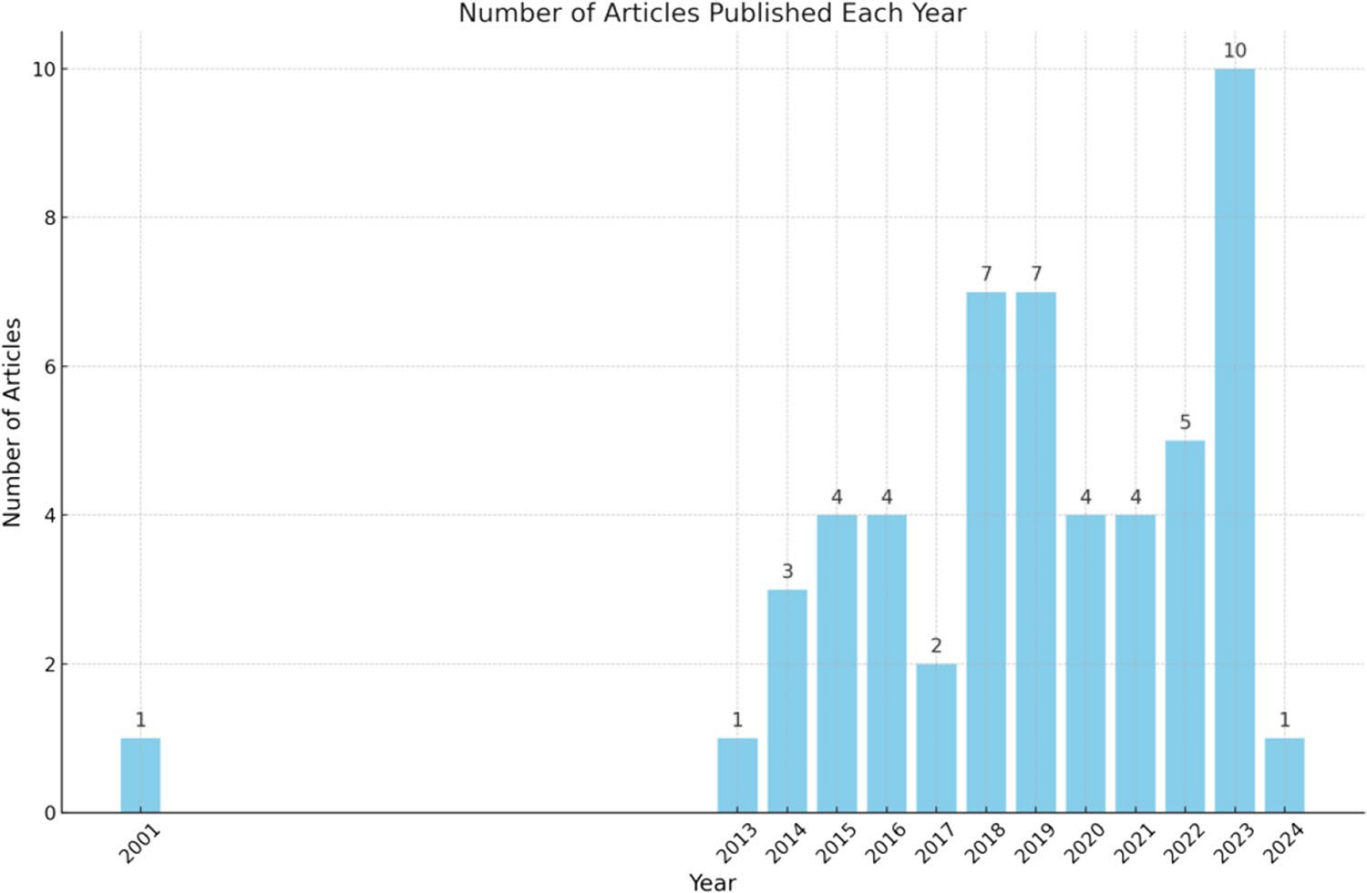
Number of articles published each year about adult ACEs screening in primary care settings

The analysis of publication years reveals a notable trend in research focused on ACE screening in primary care settings over time (See Fig. 2). There was a single study published in 2001 by Dube et al., which utilized the largest sample from the Kaiser Permanente group’s original ACEs study dataset (Felitti et al., 1998). Following this, there was a gap in publications until 2013. After 2013, the number of articles gradually increased, peaking in 2023 and reflecting a growing scholarly interest in ACE screening within primary care and its potential long-term impacts.

Among the studies included in the review, 48 studies (90.6%) used the original ACE questionnaire developed by Felitti et al. (1998). Five studies utilized the original ACE questionnaire with minor modifications and cultural adaptations to suit the needs of the specific population they were surveying. Of these five studies, three were conducted in the USA (Njorege et al., 2023; Peck et al., 2021; Rose et al., 2016), one in Iraq (Alshawi & Lafta, 2014), and one in Mexico (Moreno-Guzmán et al., 2023).

In terms of study design, the majority of the included articles (66%, *n* = 35) employed a quantitative cross-sectional approach, with studies conducted both in the United States and internationally. Quantitative longitudinal designs, including both retrospective and prospective cohort studies, represented 7.6% of the studies (*n* = 4). Only one study used a purely qualitative approach (2.5%, *n* = 1), while mixed method, quasi-experimental, and test-retest reliability designs each comprised 2.5% of the total studies (*n* = 1 for each). Additionally, explorative descriptive and pre-post intervention designs each represented 2.5% of the studies.

Regarding study purpose, feasibility studies made up 7.5% of the methodologies (*n* = 4; e.g., Glowa et al., 2016; Kalmakis et al., 2018). Quality improvement studies accounted for 5.7% (*n* = 3), while pre-post intervention designs were used in 3.8% (*n* = 2). Only 11 of the 38 studies (28.9%) conducted in the United States stated that the Spanish version of the ACEs questionnaire was readily available to non-native English speakers (Alvarez et al., 2019; Coon et al., 2021; Gebauer et al., 2015; Jelley et al., 2020; Loeb et al., 2022; Miller-Cribbs et al., 2016; Nishida, 2015; Njoroge et al., 2023; Scherrer et al., 2014; Sosnowski et al., 2023; Zak-Hunter et al., 2023).

### Demographics

In Table 1, the overall sample size and percentage distribution by gender and ethnicity were calculated from available information provided in 53 studies conducted globally. The demographic characteristics of the 42,415 adult patients screened for ACEs in primary care settings revealed that a majority were female (64.9%) and White (58.3%). Males constituted 34.5% of the sample, while other gender identities, including non-binary and undisclosed, accounted for less than 0.2%. In terms of race and ethnicity, Black/African American patients comprised 8.8%, Hispanic/Latin individuals made up 3.2%, and Asian patients represented 1.4%. Additionally, smaller proportions included American Indian/Native Alaskan (0.3%), Native American/Pacific Islander (0.09%), and multiracial (0.1%) participants. A significant portion of the sample was categorized as “Other” (14.7%), highlighting the diverse background of the patient population unrecognized. Furthermore, participants identified as Iraqi (2.3%; AlShawi & Lafta, 2014), Irish (4.81%; Sinnott et al., 2015), Bosniaks (0.9%; Musa et al., 2018), and Lebanese (0.7%; Sukkarieh et al., 2023) were included based on studies conducted in their respective countries. Notably, these four studies, along with two others conducted outside of the United States (Hardcastle et al., 2020; Maunder et al., 2019), did not report any race/ethnicity information related to their sample size. Among the U.S. studies, three reported that their samples consisted exclusively of White/Caucasian participants (Kalmakis et al., 2018; Priestley, 2023; Rose et al., 2016), while one study focused solely on Latina women (Alvarez et al., 2019), and another deliberately excluded White participants to better understand the experiences of racially and ethnically diverse groups (Zak-Hunter et al., 2023). While the Hispanic/Latinx population was primarily from studies conducted in the United States, there was also a study conducted in Mexico predominantly involving Hispanic/Latin participants (*n* = 139; Moreno-Guzmán et al., 2023). This study was the only non-English study included in this systematic review.

**Table 1:** Demographic characteristics of adult patients screened for ACEs in primary care settings (*N =* 42,415)

	*N*	%
Gender		
Female/women	27,548	64.9
Male/men	14,581	34.5
Non-binary	65	0.1
Did not disclose	21	0.05
Other	20	0.05
Reported missing	95	0.2
Not reported in the article	85	0.2
Race/Ethnicity*		
White	24,763	58.3
Black/African American	3,753	8.8
Hispanic/Latin	1,339	3.2
Asian	584	1.4
American Indian/Native Alaskan	114	0.3
Native American/Pacific Islander	40	0.09
Multiracial	44	0.1
First Nations	43	0.1
Iraqi	1,000	2.3
Lebanese	300	0.7
Irish	2,047	4.81
Bosniaks	400	0.9
Unknown	59	0.1
Other	6,234	14.7
Not reported in the article	1,777	4.2

The terminology employed to refer to gender and race/ethnicity is as used in the relevant literature

*The total number of race/ethnicity responses exceeds the actual sample size because some studies allowed participants to select multiple race/ethnicity categories. The percentages are calculated based on this higher total

In almost 70% of the studies (*n* = 37), the sample population primarily comprised adult patients from general primary care settings. However, there were also studies focused on pregnant women (*n* = 4, Fields et al., 2023; Sosnowski et al., 2023; van Roessel et al., 2021; Zak-Hunter et al., 2023), individuals with chronic pain or illness (*n* = 6, Garland et al., 2019; Gebauer et al., 2015; McSwan et al., 2023; Purkey et al., 2018a, b; Rose et el., 2016; Scherrer et al., 2014), patients with diabetes (*n* = 4, Johnston, 2020; Priestley, 2023; Strenth et al., 2022; Sukkarieh et al., 2023), individuals living with HIV (*n* = 1, Peck et al., 2021), and patients with substance use disorders (*n* = 1, Pykare and Knox, 2022) who are receiving primary care services. Additionally, 15% (*n* = 8) of the studies included in the systematic review exclusively involved female patients, whereas there were no studies conducted solely with male participants.

### Rationale for ACE Screening in Primary Care

We discovered that 44 out of 53 studies (83%) worldwide performed ACE screening solely for research purposes. In the United States, this rate is 84.2%. Based on our classification criteria, studies were categorized as “research only” if they did not provide a stated rationale for ACE screening beyond data collection. All 44 studies classified under this category lacked a clear rationale or intervention plan and were therefore categorized as “research only.”

Among the remaining studies conducted in the U.S., three indicated ACE screening as part of a quality improvement initiative (Johnston, 2020; Peck et al., 2021; Pykare & Knox, 2022), and three articles (Goldstein et al., 2019; Priestley, 2023; Vishwanath and Maxwell, 2023) indicated ACE screening as part of a trauma-informed primary care intervention plan.

Among non-U.S. studies, only one study from Australia (McSwan et al., 2023) reported implementing a quality improvement initiative. One of the eight studies conducted in Canada (van Roessel et al., 2021) indicated the recent implementation of routine ACE screening as part of a TIC intervention alongside mental health screening. Globally, only one study (United Kingdom; Hardcastle et al., 2020) stated that ACE screening was routinely performed as part of primary healthcare services in their institution.

### Adult ACE Screening Procedures in Primary Care

In our study, we examined 53 primary care studies that utilized Adverse Childhood Experiences (ACE) screening methods. The data revealed that the majority of the studies (*n* = 40, 75.5%) employed on-site screening methods. Within this category, self-reporting was the predominant method, accounting for 75% (*n* = 30) of the on-site screenings. Specifically, these self-reports were conducted in private rooms (*n* = 6, 15%), waiting rooms (*n* = 6, 15%), and locations not mentioned in the study design (*n* = 18, 45%). Staff-administered screenings made up 25% (*n* = 10) of the on-site screenings, predominantly conducted in confidential rooms (*n* = 5, 50%), waiting rooms (*n* = 1, 10%), or locations not mentioned in the study design (*n* = 4, 40%). See the Supplementary Table for details on the professional roles involved in ACE screenings within primary care settings.

Off-site screenings were utilized in 15.1% (*n* = 8) of the studies. Self-report methods accounted for 62.5% (*n* = 5) of off-site screenings and were conducted online or by mail (*n* = 5, 100%). Staff-administered screenings constituted 25% (*n* = 2), both conducted via tele-survey (100%, *n* = 2). Additionally, one study (12.5%, *n* = 1, Zak-Hunter et al., 2023) employed a mixed off-site approach that combined self-report and staff-administered methods, which included tele-surveys.

A mixed approach of both on-site and off-site screening was reported in 5.7% (*n* = 3) of the studies. One study (33.3%, *n* = 1) utilized self-reports, allowing participants to complete surveys in waiting rooms and continue at home, mailing the surveys back via pre-paid envelopes upon completion. Another study (33.3%, *n* = 1) used staff-administered screenings, where participants completed surveys in private rooms on site, and administrators followed up via phone if they needed to leave before completing the surveys. The final study in this category (33.3%, *n* = 1) combined self-report and staff-administered methods, enabling participants to complete surveys in private rooms on-site or return them via pre-paid envelopes. Participants also had the option to complete surveys through phone calls arranged by the administrators.

Two studies (3.8%, *n* = 2) did not specify the site of the screening. One study (Poole et al., 2017) used self-report ACE screenings collected online or via paper format, and the other (Young-Wolff et al., 2019) did not specify the format or administration method.

### Staff/Researcher Training and Fidelity Measures

Out of 53 articles, 10 (18.9%) reported staff or researcher training, 5 (9.4%) included measures for research fidelity, and 2 (3.8%) reported both training and fidelity measures.

Staff training in ACE screening included various approaches. One study trained primary care faculty, residents, and medical students on health issues related to post-traumatic stress disorder, ACEs, and social determinants of health in one study (Coon et al., 2021). Another study provided educational sessions on ACEs, their impact, and Trauma-Informed Care (TIC), along with instructions on using a screening tool and intervention recommendations (Johnston, 2020). Nurse Practitioner (NP) student interviewers attended training sessions covering ACE-related long-term health effects, TIC in healthcare, and mock interviewing techniques (Kalmakis et al., 2018). Research assistants received training on consent procedures, recruitment, distress referral, and data handling in one study (Miller-Cribbs et al., 2016), while all research staff were trained on interview techniques, engagement, confidentiality, and study goals in another (Njoroge et al., 2023).

Other studies reported training methods, such as interactive webinars on ACEs with staff feedback tools (Pykare and Knox, 2022), training for assistants on approaching patients and providing materials (Nishida, 2015), and informing clinicians about previous ACE research findings before screening implementation (Glowa et al., 2016). In one study, a research assistant responsible for recruitment received unspecified training (McCall-Hosenfeld et al., 2014). Another study highlighted the principal investigator’s expertise in developing trauma education curricula for healthcare professionals (Goldstein et al., 2019).

Fidelity measures included session notes to ensure adherence to protocols (Goldstein et al., 2019), weekly mentoring sessions for providers and staff (Johnston, 2020), and weekly meetings to address data collection issues and cross-checked data entry (Miller-Cribbs et al., 2016). In one study, survey administrators were nurses supervised by a research field coordinator (Musa et al., 2018), while another involved medical students supervised by faculty for respondent enrollment and questionnaire administration (Gebauer et al., 2015).

### Utilization of ACE Scores

The majority of the 53 studies analyzed aligned with a single utilization category, while four studies (*n* = 4, 7.55%; Grant, 2023; Njoroge et al., 2023; Sukkarieh et al., 2023; Zak-Hunter et al., 2023) utilized ACE scores in multiple ways. Of the total studies, 20 (37.7%) used ACE scores in correlation/regression analyses with disease diagnosis, and 11 (20.8%) examined correlations with health risk behaviors. Eight studies (15.1%) utilized ACE scores to identify healthcare access, nonattendance, literacy, referral for counseling services, protective factors, and/or social determinants of health. Three studies (5.7%) applied ACE scores to identify patterns within specific groups such as pregnant women, Latinos, or patients with type 2 diabetes. An additional three studies (5.7%) used ACE scores to facilitate discussions between medical providers and patients, while three studies (5.7%) used ACE scores as eligibility criteria for interventions. One study (1.9%) used ACE scores to assess screening-related preferences. Among the four studies that applied ACE scores in multiple ways, two combined analyses with disease diagnosis and healthcare access or social determinants, while the other two included analyses with health risk behaviors, disease diagnosis, healthcare access, and group-specific ACE patterns. For more details, please refer to the Supplementary file.

### Clinical Responses for Positive ACE Scores

Out of the 53 studies reviewed, 21 (39.6%) mentioned offering at least one type of clinical response following ACE screening, categorized into resources provided, referrals made, and direct interventions implemented. Specifically, 11 (20.7%) reported providing educational materials and information handouts to patients, while 15 studies (28.3%) documented referrals to behavioral health services, counseling, or social workers. Direct interventions, such as onsite counseling and crisis intervention services, were implemented in 14 studies (26.4%). Three studies (5.7%) reported providing all three types of responses (Enochs, 2019; Goldstein et al., 2019; Vishwanath and Maxwell, 2023). Additionally, three studies (5.7%) provided both resources and referrals, seven studies (13.2%) provided both referrals and interventions, and three studies (5.7%) reported both resources and interventions. However, 32 studies (60.4%) did not report any follow-up clinical services after ACE screening. For detailed information on each clinical response identified through this review, refer to the Supplementary file.

## Discussion

The seminal research conducted by Felitti et al. (1998) established a significant correlation between childhood adversities and subsequent well-being and health outcomes, thereby catalyzing the development of a novel research domain spanning various stages of life, diverse social contexts, and multiple institutional settings. Despite the extensive research over the past two decades, the clinical utilization of ACEs screening in primary healthcare, particularly for adult patients, has received less attention (Finkelhor, 2018; Ford et al., 2019; Oral et al., 2016). This oversight is significant because primary care serves as a crucial entry point for addressing the long-term impacts of ACEs, particularly in the U.S., where it functions as a de facto mental health system (Moise et al., 2021). Effective ACEs screening in primary care can help providers assess the level of care needed and connect patients to appropriate services, potentially mitigating the adverse health effects of ACEs across the lifespan (Kalmakis & Chandler, 2015; Larkin et al., 2014; Pachter et al., 2017). However, for ACEs screening to be beneficial, it must be integrated into a well-established trauma-informed care system, which includes provider training, sensitive screening protocols, and a robust referral network to support patients post-screening (Machtinger et al., 2015; Purkey et al., 2018a, b; Substance Abuse and Mental Health Services Administration, 2014). This review aimed to address the gap in the literature by systematically addressing the rationale, methodology, and outcomes of ACE screening in primary care settings, shedding light on the significance of these screenings and the current state of our response to their outcomes.

### ACEs – Why Do We Screen?

According to our research, the primary motivation for screening adults in primary care is to gather data (research) rather than to address individual health concerns. Specifically, 44 out of the 53 studies reviewed (approximately 83%) conducted ACEs screening primarily for research purposes. This emphasis on data collection over direct clinical application raises important ethical considerations, particularly when screening does not lead to follow-up support or interventions for patients with high ACE scores.

However, it is crucial to acknowledge that not all studies neglected to provide resources or interventions. Our findings show that 21 studies (39.6%) reported offering at least one type of clinical response following ACEs screening. These responses included providing educational materials, making referrals to behavioral health services, and implementing direct interventions such as onsite counseling and crisis intervention services. Despite these efforts, a significant proportion of studies (32 out of 53, or 60.4%) did not report any follow-up clinical services after ACEs screening. This gap suggests that while some researchers are integrating patient care into their study designs, a majority may not be adequately addressing the immediate needs of patients identified as having high ACE scores.

The ethical implications of screening without appropriate follow-up are well-documented in the literature. Inquiries about ACEs are inherently sensitive and have the potential to retraumatize individuals in the absence of effective management of such dialogues (Kia-Keating et al., 2019). To mitigate these risks, it is essential that ACEs screening is accompanied by adequate training for medical providers in trauma-informed care practices (Schulman & Maul, 2019). Such training equips providers with the skills to manage sensitive disclosures and to offer appropriate resources or referrals.

Our review shows that a significant proportion of adult patients screened for ACEs in primary care settings are White/Caucasian (58.3%) and female (64.9%), with minority communities underrepresented, which is concerning, given that minority and underrepresented groups often report higher ACE scores, which are linked to greater health risks and barriers to care (Font & Maguire-Jack, 2016; Swedo et al., 2023). The predominance of White participants suggests that racial, ethnic, and sexual minorities might not be adequately included in screening efforts, raising questions about whether those most in need are being identified and served. Although our global review included diverse demographics, U.S. studies largely failed to adequately represent minority groups, with a few exceptions like Alvarez et al. (2019), which focused exclusively on Latina women, and Zak-Hunter et al. (2023), which deliberately excluded White participants to highlight the experiences of racially and ethnically diverse groups. This trend underscores the need for more inclusive research and culturally responsive care models to better serve these populations.

### ACEs – How Do We Screen?

Our analysis identified considerable variability in the administration of ACEs screening in adult primary care, including differences in location (onsite 77.4% vs. offsite 16.9%), screening environment (private room 34.1% vs. waiting room 21.9%), and mode of screening (self-report 71.7% vs. staff-administered 26.4%). Notably, a substantial proportion of publications (45.3%) did not specify the location where onsite ACEs screenings were conducted. This lack of detailed reporting highlights a gap in the literature, underscoring the need for greater transparency and consistency in ACEs screening to help identify best practices for various settings and populations, ultimately enhancing patient care.

The variability in ACE screening methods has significant implications for primary care. Staff-administered screenings ensure thoroughness and provide immediate support but are resource-intensive and may not be feasible in all settings. Self-administered screenings, which were the most common method in our review, are cost-effective and suitable for high-volume practices. However, the impact of screening mode on data accuracy and patient outcomes remains unclear. While limited research directly compares provider-delivered screening to self-administered methods, some studies suggest that provider-delivered screening may enhance patient comfort and facilitate meaningful discussions (Goldstein 2016). Patients generally report being comfortable when clinicians inquire about ACEs and perceive their providers as competent in addressing trauma-related issues, which can strengthen the patient-provider relationship and support trauma-informed care (Goldstein 2016).

Conversely, barriers such as lack of time, insufficient training, and concerns about retraumatization can hinder providers’ ability to effectively deliver ACEs screening (Clark & Jones, 2022; Nutting et al., 2023). Providers often report low confidence in screening for ACEs and following up with patients, highlighting the need for enhanced training and systemic support (Nutting et al., 2023). Addressing these barriers is crucial, as provider-delivered screening, when conducted within a trauma-informed framework, has the potential to improve patient engagement and facilitate appropriate interventions.

The screening environment also plays a critical role. Only 30.9% of onsite screenings were conducted in private settings, which are essential for maintaining confidentiality and fostering open communication. From a trauma-informed care perspective, ensuring a safe and supportive environment is crucial for effective screening (Substance Abuse and Mental Health Services Administration [SAMHSA], 2014). Patients with elevated ACEs or PTSD symptoms may experience increased stress during medical visits, and provider behaviors that promote safety and trust can mitigate this stress (Loeb et al., 2022).

Trauma-informed care (TIC) is widely recognized as a fundamental basis for ACEs screening (Substance Abuse and Mental Health Services Administration, 2014). The literature consistently emphasizes that ACEs screening should only be conducted when the necessary preparations have been thoroughly implemented within the institution and among staff members (Albaek et al., 2018; Gopal et al., 2023; Leasy et al., 2019; Raja et al., 2015; Watson, 2019). This approach is essential because addressing ACEs effectively requires a well-coordinated team effort (Valeras et al., 2019), which is achieved by making institutions more trauma-informed through comprehensive training provided to all team members (Leasy et al., 2019). However, our review revealed that only 10 (18.9%) out of 53 studies reported providing any form of training to researchers or medical providers conducting ACEs screenings. Additionally, only 5 studies documented assessment fidelity. This low prevalence of training and fidelity documentation across studies raises significant concerns. Without adequate training, there is an increased risk of retraumatizing patients or mishandling the sensitive information gathered during ACEs screening, which could lead to inconsistent practices and variability in the quality of care, ultimately undermining the potential benefits of ACEs screening in primary care settings (Raja et al., 2015; Zarnello, 2023).

To mitigate these risks and enhance the effectiveness of ACEs screening, clinical settings should adopt organization-tailored trauma-informed approaches that prepare staff to handle the complexities of ACEs. Providing adequate training can improve providers’ confidence in conducting ACEs screening and following up with patients (Nutting et al., 2023), facilitating meaningful conversations and appropriate interventions. Addressing systemic barriers such as time constraints and lack of resources is also essential for integrating provider-delivered ACEs screening into routine practice (Clark & Jones, 2022). One effective strategy could be to include a clinical supervisor on the healthcare team. This supervisor could drive positive changes by mastering the culture of the clinic and understanding patient needs, providing essential training on ACEs and trauma-informed practices, offering support in challenging areas, enhancing patient outcomes without disrupting appointment flow, and introducing clinic-specific interventions while ensuring ongoing evaluation throughout the process.

### ACEs – What Do We Do with Primary Care Patients’ ACE Scores?

Understanding how ACE scores are applied is critical to informing clinical practices and public health policies. Our review identified seven primary ways ACE scores are utilized across the included studies: (1) analyzing correlations with health risk behaviors, (2) disease diagnosis, (3) assessing healthcare access, nonattendance, literacy, referrals, protective factors, and social determinants, (4) identifying specific groups’ ACE patterns, (5) informing healthcare visit discussions, (6) serving as eligibility criteria for interventions, and (7) determining ACE screening preferences.

The most common application of ACE scores in the reviewed studies was for correlation/regression analyses with disease diagnosis and health risk behaviors, suggesting a strong focus on understanding the direct health impacts of ACEs. However, only a small number of studies reported using ACE scores during patient visits or as criteria for interventions, which highlights a potential underutilization of these scores in direct patient care. In other words, while we—as researchers—continue to deepen our understanding of ACEs-related health outcomes based on participants’ ACE scores, we seem to fall relatively short in providing proactive support to these participants in response to their scores. This issue is further complicated by the lack of clarity on the appropriate interventions for asymptomatic patients with high ACE scores, as highlighted by Campbell (2020). The ambiguity surrounding intervention strategies for such patients underscores the need for more comprehensive guidelines that can inform clinical decision-making.

This need for a more systematic and proactive approach to utilizing ACE scores is further emphasized by the findings of a scoping review on ACE screenings in preventive medicine settings (Mishra et al., 2023). The study highlights that while ACE screenings can be integrated into routine clinical workflows without significant disruption, the success of such integration largely depends on adequate staff training and institutional support. Furthermore, the review underscores the importance of a trauma-informed approach to ensure that patients feel safe and supported during screenings. However, the study also identifies significant barriers, including time constraints, lack of resources, and concerns about retraumatization, which can hinder the effective use of ACE scores in patient care (Mishra et al., 2023). These challenges mirror the underutilization of ACE scores in providing direct clinical care or interventions, as identified in our review, reinforcing the need for robust research and the development of standardized guidelines to optimize the use of ACE scores in clinical practice.

### ACEs – Where Are We at the End of the Day?

The trajectory of research publications on adult ACEs screening in primary care settings shows a growing body of research and increasing focus on the role of primary care in addressing the long-term impacts of childhood adversity. After the early work by Dube et al. in 2001, research activity began to rise steadily from 2013 onward, indicating heightened awareness of the need for ACEs screening within primary care. This overall increase points to a sustained and growing renewed interest in ACEs screening as the healthcare field continues to recognize its importance in addressing patients’ holistic health needs.

Despite this growing body of research, the follow-up actions and clinical responses remain inconsistent. Our analysis revealed significant gaps in the specific clinical services provided following the identification of high ACE scores in adult patients in primary care settings. The findings suggest that despite some promising practices, there is a significant gap in consistent and comprehensive trauma-informed care. Our review found that while some studies reported providing educational resources (20%) and making referrals to behavioral health services (28%) or offering direct interventions (26%), more than half (61%) did not report any follow-up clinical services. The provision of educational resources, while beneficial in raising awareness, often lacks the necessary follow-through to ensure that patients can access further help. The same issue was consistent with the studies that referred participants with positive ACE scores to either onsite or offsite behavioral or mental health specialists. Many of these studies did not follow up to see whether participants actually engaged with the referred specialists. This raises concerns about the effectiveness of simply providing information or referrals without ensuring that patients receive the necessary follow-up support.

Furthermore, a systematic review (Strauch et al., 2022) revealed significant gaps in how ACEs are communicated in primary care, noting that discussions often focus more on screening than on meaningful conversations about trauma. This lack of thorough communication limits the effectiveness of interventions and misses critical opportunities for early support. In conclusion, while the rise in research on adult ACEs screening in primary care is a positive development, there is still much work to be done to translate these findings into consistent, impactful practices. This will require a more structured approach to follow-up care, the exploration of diverse interventions, and the enhancement of communication strategies to ensure that screening leads to meaningful, trauma-informed support for patients.

### Limitations

The present review was conducted with a systematic approach to searching and extracting data, utilizing explicit inclusion criteria, which is a notable strength of this review. However, it is important to acknowledge the limitations associated with the study. To ensure consistency in our analysis, we included only studies that used the original, widely used ACEs questionnaire published by Felitti et al. (1998) as one of the inclusion criteria; we did not include studies that used other forms of ACEs screeners. However, studies that reported cultural adaptations of certain items while adhering to the original questions were also included. Our rationale for doing this is that ACEs applications in some different versions require a certain utilization and setting due to the nature of the questionnaire used. For example, the ‘ACEs Questionnaire for Adults’ (California Surgeon General’s Clinical Advisory Committee, n.d.) requires self-reporting because the respondent was asked to list only their total ACE scores without marking the adversities they experienced. This limitation may create a barrier in terms of our systematic review in generalizability. Lastly, only studies examining multiple ACEs were included in the review; if studies examining only one type of ACE (for example, domestic violence) had been included, the results of the review might have been different.

## Conclusion

Strong evidence connects ACE scores to adult health problems (Felitti et al., 1998), highlighting the need for enhanced ACEs detection, interpretation, and treatment in healthcare settings (Burke et al., 2011; Weinreb et al., 2010). To the best of our knowledge, there is no comprehensive systematic review in the literature reporting why and how ACEs screening is performed with adult patients in primary care contexts and what clinical services are provided to adult patients based on the scores obtained. Our global review revealed that routine ACEs screening is rare, with only one UK study reporting such practice, while nearly 85% of studies primarily screened for research purposes. Additionally, most ACEs screenings were conducted onsite, yet only a minor percentage took place in confidential settings, and almost half were self-administered by patients. Notably, ACEs training and screening fidelity rates were low. Future research should identify optimal utilization practices from both provider and patient perspectives and explore how ACE scores should be interpreted and what interventions are most appropriate for different scores.

## Supplementary Information

Below is the link to the electronic supplementary material.

ESM 1 (DOCX 53.6 KB)

## Data Availability

The data for this systematic review consists of published articles and online accessible dissertations, which are available through their respective journals and databases. A list of all sources included in the review is provided in the manuscript, and further details can be made available upon request.
