# Supplementary material for: Adverse Childhood Experiences (ACEs) Screening in Primary Care Settings for Adults: A Systematic Review
Source: J Child Adolesc Trauma. 2025 Feb 12;18(2):377–93. doi: 10.1007/s40653-025-00691-4 (PMC12129883; doi:10.1007/s40653-025-00691-4)
Supplement: Supplementary file 1 — (DOCX 53.6 KB) [file 40653_2025_691_MOESM1_ESM.docx]

**Supplementary Table**

*Characteristics of all studies included in the review.*

|  |  |  |  |  |  |  |  | **Clinical Services** |  |  |
| --- | --- | --- | --- | --- | --- | --- | --- | --- | --- | --- |
| **Citation** | **n size/ Country,**  **state** | **Study Design** | **Sample  (a) Race, % (b) Ethnicity, % (c) Gender, %** | **ACEs and # of items** | **Procedure (a) Where  (b) Administrator (c) Profession**  **(d) Translated ACEs for non-natives** | **Training and Fidelity  (a) Staff/ Researcher training (b) Fidelity measures** | **How are ACEs used?** | **Resources** | **Referrals** | **Interventions** |
| AlShawi & Lafta, 2014 | 1000/Iraq, Baghdad | Cross-sectional study | **(a)** - **(b)** - **(c)** Female, 58.3%; Male, 41.7% | ACE-IQ^1^ 9 Items | **(a)** Closed room for privacy (on-site) **(b)** Self-report  **(c)** NA  **(d)** No | **(a)** ✗ **(b)** ✗ | **(1)** Used in analysis - Correlation/Regression analysis with health risk behaviors | ✗ | ✗ | ✗ |
| Alvarez et al., 2019 | 235/USA, Maryland | Cross-sectional study | **(a)** - **(b)** Latina, 100% **(c)** Female, 100% | ACE^2^  10 items | **(a)** Not mentioned (on-site: electronically) **(b)** Self-report (Staff assisted if needed) **(c)** PI & Research assistant (translation)  **(d)** Yes | **(a)** ✗ **(b)** ✗ | **(1)** Used in analysis - Correlation/Regression analysis with health risk behaviors | ✗ | ✗ | ✗ |
| Chapman et al., 2022 | 32/USA, Oregon | Mixed methods | **(a)** White 75%; African American, 9.3%; American Indian/Native Alaskan, 3.2% **(b)** Latinx, 12.5% **(c)** Female, 75%; Male, 25% | ACE^2^ 10 items | **(a)** Private room in a community workspace (on-site & over the phone) **(b)** Staff-Administered  **(c)** Research Team **(d)** No | **(a)** ✗ **(b)** ✗ | **(3)** Identify healthcare access, nonattendance, literacy, referral for counseling services, protective factors, and/or social determinants of health | ✗ | ✗ | ✗ |
| Chuks-orji, 2019  (Dissertation) | 122/USA, California | Pre- and post- intervention design | Pre - Post intervention: **(a)** White, 25.9 - 32.4%; African American, 3.7 -22.1%; Asian, 5.6 - 7.4%; Other, 9.3 - 7.4% **(b)** Latino, 61.1 - 32.4% **(c)** Female, 59.3 - 60.3%; Male, 40.7 - 39.7% | ACE^2^ 10 items | **(a)** Not mentioned (on-site) **(b)** Staff Administered  **(c)** Nurse Practitioner **(d)** No | **(a)** ✗ **(b)** ✗ | **(3)** Identify healthcare access, nonattendance, literacy, referral for counseling services, protective factors, and/or social determinants of health | ✗ | Patients with ACE score > 1 were given a referral for BH services | NP reviewed the questionnaires and interpreted patients’ scores, discussed ACE score, and options |
| Coon et al., 2021 | 354/USA, Oklahoma | Cross-sectional study | **(a)** White, 65 %; Black, 21%; Hispanic, 8%; Asian, 1%; Amer. Indian/Alaska Native, 12%; Native Amer./Pacific Isl, 1% **(b)** - **(c)** Female, 75%; Male, 25% | ACE^2^ 10 items | **(a)** Not mentioned (On-site) **(b)** Self-report (Staff assisted if needed) **(c)** Research team **(d)** Yes | **(a)** Researchers trained primary care faculty, residents, and medical students to address health issues associated with PTSS, ACE, and SDH. **(b)** ✗ | **(3)** Identify healthcare access, nonattendance, literacy, referral for counseling services, protective factors, and/or social determinants of health | ✗ | ✗ | ✗ |
| Dube at al., 2001 | 17337/USA, California | Retrospective cohort study | **(a)** White, 75% **(b)** - **(c)** Female, 54%; Male, 46% | ACE^2^ 10 items | **(a)** NA (off-site) **(b)** Self-report **(c)** NA **(d)** No | **(a)** ✗ **(b)** ✗ | **(1)** Used in analysis - Correlation/Regression analysis with health risk behaviors | Detailed information about ACEs and household dysfunction within the survey pack | ✗ | ✗ |
| Enochs, 2019  (Dissertation) | 8 Provider; 30 Patients/ USA, California | Quasi-experimental | **(a)** Caucasian patients, 36.7%; African American/Black patients, 3.3%; Asian/Pacific Islander patients, 3.3%  **(b)** Hispanic/Latino Patients, 56.7% **(c)** Provider Female, 75%; Provider Male, 25%; Patient Female; 83.3%; Patient Male, 16.7% | ACE^2^ 10 items | **(a)** Private office room **(b)** Self-report **(c)** NA **(d)** No | **(a)** ✗ **(b)** ✗ | **(5)** For medical provider to discuss ACEs score results during visit | ACE information handout, and resource information was given to patients | Referrals for counseling | During patients' visits, providers discussed ACEs result with their patients |
| Fields et al., 2023 | 133/USA, South Central | Prospective Study | **(a)** African American/Black patients, 28%; Native American, 17.8%; Hispanic, 13.6%; White, 39.4%  **(b)** Hispanic/Latino Patients, 13.6% **(c)** Female, 100% | ACE^2^ 10 items | **(a)** Online (on-site)  **(b)** Self-report **(c)** NA  **(d)** No | **(a)** ✗ **(b)** ✗ | **(3)** Identify healthcare access, nonattendance, literacy, referral for counseling services, protective factors, and/or social determinants of health | ✗ | ✗ | ✗ |
| Frampton et al., 2018 | 284/Canada, Alberta | Test-retest reliability | **(a)** Caucasian, 85.2%; Minority, 14.8% **(b)** - **(c)** Female, 74.6%; Male, 25.4% | ACE^2^ 10 items | **(a)** Online (off-site) **(b)** Self-report **(c)** NA  **(d)** No | **(a)** ✗ **(b)** ✗ | **(2)** Used in analysis - Correlation/Regression analysis with disease diagnosis | ✗ | ✗ | ✗ |
| Gaba et al., 2023 | 131/USA, New York | Cross-sectional study | **(a)** Native American, 2.5%; Asian, 2.5%; Black/African American, 35.5%; White, 57.9%  **(b)** Hispanic/Latino, 15.6%; Not Hispanic/Latino, 78.1% **(c)** Female, 56.6%; Male, 41.1%; Missing, 2.3% | ACE^2^  10 items | **(a)** Waiting room **(b)** Self-report **(c)** NA  **(d)** No | **(a)** ✗ **(b)** ✗ | **(7)** Identify ACEs screening related preferences | Participants received information for external behavioral health resources. | Behavioral health clinicians were available to meet with patients during the study period should a participant become acutely distressed. | ✗ |
| Grant, 2023 (Dissertation) | 349/USA, Missouri | Retrospective case-control stud | **(a)** -  **(b)** Caucasian/European, 79.9%; Asian, 4%; Black/African American, 11.5%; White, 57.9%; Hispanic/Latino, 1.7%; American Indian, 1.1%; Other, 1.4% **(c)** Female, 71.3%; Male, 28.7%; Missing, 2.3% | ACE^2^ 10 items | **(a)** Off-site (online) **(b)** Self-report **(c)** NA  **(d)** No | **(a)** ✗ **(b)** ✗ | **(2)** Used in analysis - Correlation/Regression analysis with disease diagnosis  **(3)** Identify healthcare access, nonattendance, literacy, referral for counseling services, protective factors, and/or social determinants of health | Participants received crisis hotline resources | ✗ | ✗ |
| Garland et al., 2019 | 36/USA, Utah | Cross-sectional study | **(a)** Caucasian, 88.9%; African American, 2.8%; Latino, 2.8%; Asian, 2.8%; American Indian, 2.8% **(b)** - **(c)** Female, 100% | ACE^2^ 10 items | **(a)** Not mentioned (on-site) **(b)** Self-report **(c)** NA **(d)** No | **(a)** ✗ **(b)** ✗ | **(2)** Used in analysis - Correlation/Regression analysis with disease diagnosis | ✗ | ✗ | ✗ |
| Gaska & Kimerling, 2018 | 6212/USA, national | Cross-sectional study | **(a)** White, 65%; Black/African American, 28%; Other, 8% **(b)** - **(c)** Female, 100% | ACE^2^  7 items | **(a)** NA (telephone survey) **(b)** Staff-administered **(c)** Not mentioned **(d)** No | **(a)** ✗ **(b)** ✗ | **(4)** Identify certain groups' ACEs patterns | ✗ | ✗ | ✗ |
| Gebauer et al., 2015 | 213/USA, Texas | Cross-sectional study | **(a)** White, 46%; Non-white, 54% **(b)** - **(c)** Female, 66%; Male, 34% | ACE^2^ 17 items | **(a)** Not mentioned (On-site) **(b)** Self-report (staff assisted if needed) **(c)** Medical students  **(d)** Yes | **(a)** ✗ **(b)** Medical students, supervised by faculty, enrolled respondents and administered questionnaires. | **(2)** Used in analysis - Correlation/Regression analysis with disease diagnosis | ✗ | ✗ | ✗ |
| Glowa et al., 2016 | 111/ USA, Vermont | Feasibility Study | **(a)** - **(b)** - **(c)** Female, 61%; Male, 39% | ACE^2^ 10 items | **(a)** Private office room (On-site) **(b)** Staff administered **(c)** Nurse **(d)** No | **(a)** All clinicians were informed about previous ACE research  findings before the study. **(b)** ✗ | **(5)** For medical provider to discuss ACEs score results during visit | Clinicians discussed the issues for patients having any ACE risk | ✗ | Nursing staff provided the ACE questionnaires, and clinicians viewed them during the office visit. |
| Goldstein, 2016  (Dissertation) | 152/USA, California | Cross-sectional study | **(a)** White, not Hispanic 7.2%; Other 11.8% **(b)** Hispanic, 63.2% **(c)** Female, 64.5%; Male, 34.2% | ACE^2^ 10 items | **(a)** Not mentioned (On-site) **(b)** Self-report (Staff assisted if needed) **(c)** PhD candidate in nursing science  **(d)** No | **(a)** ✗ **(b)** ✗ | **(2)** Used in analysis - Correlation/Regression analysis with disease diagnosis | ✗ | Behavioral health counselors were available onsite if a study participant became acutely distressed | ✗ |
| Goldstein et al., 2019 | 40/USA, Wisconsin | Feasibility Study | **(a)** Black/African American, 92.5%; Non-Black/African American, 7.5% **(b)** - **(c)** Female, 67.5%; Male, 32.5% | ACE^2^ 10 items | **(a)** Clinic waiting room **(b)** Staff-administered **(c)** PI & Research assistant (translation) **(d)** No | **(a)** Principal Investigator has expertise developing trauma education curriculum for health care professionals.  **(b)** Fidelity to the protocol checked by session notes written by the investigator | **(6)** As an eligibility criterion for an intervention | ✗ | Individuals who expressed interest were introduced to an onsite counselor | Trauma-informed care intervention (Screening for ACEs, assessing health risk behavior, motivating changes in health behaviors - 2 sessions) |
| Hardcastle et al., 2020 | 763/UK, England, and Wales | Cross-sectional study | **(a)** - **(b)** - **(c)** Female, 61.9%; Male, 38.1% | ACE^2^ 10 items | **(a)** Waiting room **(b)** Self-report **(c)** NA **(d)** No | **(a)** ✗ **(b)** ✗ | **(2)** Used in analysis - Correlation/Regression analysis with disease diagnosis | ✗ | ✗ | ✗ |
| Hill, 2015  (Dissertation) | 134/USA, California | Cross-sectional study | **(a)** non-Latino White, 32%; African American, 30%; Latino, 16%; Asian-Pacific Islander, 10%; Native American, 2%; Multicultural, 10% **(b)** -  **(c)** Female, 53%; Male, 47% | ACE^2^ 10 items | **(a)** Private room / telephone survey **(b)** Self/staff-administered **(c)** NA  **(d)** No | **(a)** ✗ **(b)** ✗ | **(4)** Identify certain groups' ACEs patterns | ✗ | Participants who reported being upset due to survey were offered onsite mental health services | The ACEs items were presented in a non-threatening, non-judgmental format |
| Jelley et al., 2020 | 354/USA, Oklahoma | Cross-sectional study | **(a)** White, 65%; African American, 21%; Asian, 1%; American Indian/Alaska Native, 12%; Native American/Pacific Islander, 1% **(b)** Hispanic, 8% **(c)** Female, 75%; Male, 25% | ACE^2^  10 items | **(a)** Waiting Area and/or exam room **(b)** Self-report (staff assisted if needed) **(c)** Graduate research assistant  **(d)** Yes | **(a)** ✗ **(b)** ✗ | **(3)** Identify healthcare access, nonattendance, literacy, referral for counseling services, protective factors, and/or social determinants of health | ✗ | ✗ | ✗ |
| Johnston, 2020 (Dissertation) | 16/USA | Quality improvement pilot study | **(a)** Not specified^7^ **(b)** Not specified^7^ **(c)** Not specified^7^ | ACE^2^ 10 items | **(a)** Not mentioned  (on-site) **(b)** Self-report (staff assisted if needed) **(c)** Nurse  **(d)** No | **(a)** An educational session on ACEs, their impact, and TIC for staff, including a screening tool and intervention recommendations.  **(b)** The project included weekly mentoring for providers and clinic staff. | **(6)** As an eligibility criterion for an intervention | ✗ | Two of the 16 patients were offered mental health referrals due to their ACE scores of 4 or more but declined. | Providers utilized trauma-informed care with those who screened positive (4 or more ACEs score) |
| Kalmakis et al., 2018 | 71/USA, Massachusetts | Feasibility Study | **(a)** White European, 100% **(b)** -  **(c)** Female, 69%; Male, 31% | ACE^2^  19 items | **(a)** Not mentioned (On-site) **(b)** Staff administered **(c)** NP student **(d)** No | **(a)** NP student interviewers attended two 2-hour sessions on ACEs' long-term health effects, TIC in healthcare, and mock interviewing to learn asking about ACEs.  **(b)** ✗ | **(6)** As an eligibility criterion for an intervention | ✗ | Patient were offered referral to on-site counseling, the clinic NP, other community services | Interviewers responded with compassion, acknowledged the shared experiences (Three-phase approach to ACE screening) |
| Le et al., 2017 | 348/Canada, Toronto | Cross-sectional study | **(a)** White, 72.4%; Asian, 13.5%; Other, 14.1% **(b)** - **(c)** Female, 60%; Male, 40% | ACE^2^ 17 items | **(a)** Not mentioned (on-site) **(b)** Self-report **(c)** NA **(d)** No | **(a)** ✗ **(b)** ✗ | **(1)** Used in analysis - Correlation/Regression analysis with health risk behaviors | ✗ | ✗ | ✗ |
| Le et al., 2021 | 351/Canada, Toronto | Cross-sectional study | **(a)** White, 66.4%; Black, 5.1%; Asian, 13.3%; Other, 12.8%; Missing, 2.3% **(b)** -  **(c)** Cisgender Female, 68.7%; Cisgender Male, 28.1%; Trans Male, 0.3%; Gender non-binary, 0.9%; Other/prefer not to answer, 2.1% | ACE^2^ 10 items | **(a)** Waiting area (On-site) **(b)** Self-administrated **(c)** Not mentioned **(d)** No | **(a)** ✗ **(b)** ✗ | **(1)** Used in analysis - Correlation/Regression analysis with health risk behaviors | ✗ | ✗ | ✗ |
| Loeb et al., 2022 | 302/USA, Colorado | Cross-sectional study | **(a)** Caucasian/White, 35.8%; Black/African American, 16.9%; Unknown/Other, 33.8%; Asian, 1.3%; Native Hawaiian/Pacific Islander, 0.3%; Multiple Races, 2% **(b)** non-Hispanic, 48%; Hispanic, 47.7%; Unknown/Other, 4.3% **(c)** Female, 62.9%; Male, 32.5%; Trans, 3.6%; Unknown, 1% | ACE^2^ 10 items | **(a)** Private room **(b)** Self-report (Staff assisted if needed) **(c)** Research Assistant **(d)** Yes | **(a)** ✗ **(b)** ✗ | **(2)** Used in analysis - Correlation/Regression analysis with disease diagnosis | A resource sheet containing service information for additional support and treatment. | If survey questions led stress for patients, a licensed on-site BH provider was available for consultation. | ✗ |
| Lynch et al., 2013 | 801/USA, Northeast | Cross-sectional study | **(a)** Black, 84.6%; White, 6.1%; Other, 9.3% **(b)** -  **(c)** Female, 80.8%; Male, 19.2% | ACE^2^ 10 items | **(a)** Not mentioned (On-site) **(b)** Self-report **(c)** NA  **(d)** No | **(a)** ✗ **(b)** ✗ | **(2)** Used in analysis - Correlation/Regression analysis with disease diagnosis | ✗ | ✗ | ✗ |
| Maunder et al., 2019 | 286/Canada, Toronto | Cross-sectional study | **(a)** - **(b)** - **(c)** Female, 55.5%; Male, 44.1%; Other, 0.3% | ACE^2^ 10 items | **(a)** Mixed – on site waiting room or off site (home) **(b)** Self-report **(c)** NA **(d)** No | **(a)** ✗ **(b)** ✗ | **(2)** Used in analysis - Correlation/Regression analysis with disease diagnosis | ✗ | ✗ | Study team developed questionnaire to assess behavior change plans and which interventions they would consider - Then, researchers measured the stage of change. |
| McCall-Hosenfeld et al., 2014 | 597/USA, Massachusetts | Cross-sectional study | **(a)** Black/African American, 60.8%; White, 17.3%; Other, 11.7%; Missing, 0.3% **(b)** Hispanic/Latino, 9.9% **(c)** Female, 59%; Male, 41% | ACE^2^  10 items | **(a)** Not mentioned (On-site) **(b)** Staff Administered **(c)** Not mentioned **(d)** No | **(a)** A training (not specified) provided to research assistant who was responsible for recruitment. **(b)** ✗ | **(2)** Used in analysis - Correlation/Regression analysis with disease diagnosis | ✗ | ✗ | ✗ |
| McSwan et al., 2023 | 39/Australia, Queensland | Feasibility Study | **(a)** White, 84.6%; Non-white, 15.4% **(b)** - **(c)** Female, 71.8%; Male, 28.2% | ACE^2^  10 items | **(a)** Not mentioned (on-site) **(b)** Self-report **(c)** NA **(d)** No | **(a)** ✗ **(b)** ✗ | **(1)** Used in analysis - Correlation/Regression analysis with health risk behaviors | ✗ | ✗ | ✗ |
| Miller-Cribbs et al., 2016 | 354/ USA, Oklahoma | Cross-sectional study | **(a)** White, 93%; Non-white, 7% **(b)** - **(c)** Female, 75%; Male, 25% | ACE^2^ 10 items | **(a)** Not mentioned (On-site) **(b)** Self-report (Staff assisted if needed) **(c)** Interdisciplinary research team members **(d)** Yes | **(a)** Research assistants received training on interviewing, consent procedures, participant recruitment, distress referral, and data handling.  **(b)** Weekly meetings addressed data collection issues and data entry was cross-checked for accuracy | **(3)** Identify healthcare access, nonattendance, literacy, referral for counseling services, protective factors, and/or social determinants of health | Patients were given a list of community resources for health and mental health services, childcare, food, clothing, utilities, shelter, transportation, substance abuse, and crisis intervention services. | If the patient was distress, the interviewer contacted the onsite social worker to assess immediate needs | The research coordinator conducted a follow up telephone call to the participant within 1-3 days. |
| Moreno-Guzmán et al., 2023 | 139/Mexico, Michoacán | Cross-sectional study | **(a)** - **(b)** Hipanic/Latino/ Mestizo, 66.8%; Indigenous, 1.6%; White, 1.6%; Not declared 2.1% **(c)** Female, 58.3%; Male, 41.7% | ACE-IQ^3^ 43 Items | **(a)** In a private room  **(b)** Self-report  **(c)** NA **(d)** No | **(a)** ✗ **(b)** ✗ | **(2)** Used in analysis - Correlation/Regression analysis with disease diagnosis | ✗ | ✗ | ✗ |
| Muench et al., 2018 | 180/USA, Oregon | Cross-sectional study | **(a)** White, 86.9%; Black, 3.6%; Asian or Pacific Islander, 2.4%; Multiracial, 7.1% **(b)** Hispanic, 2.4%; Not Hispanic, 97.6% **(c)** Female, 71.4%; Male, 28.6% | ACE^2^ 10 items | **(a)** Mailed surveys (off-site) **(b)** Self-report **(c)** NA **(d)** No | **(a)** ✗ **(b)** ✗ | **(4)** Identify certain groups' ACEs patterns | ✗ | ✗ | ✗ |
| Musa et al., 2018 | 400/Federation of Bosnia and Herzegovina | Cross-sectional study | **(a)** - **(b)** - **(c)** Female, 58.5%; Male, 41.5% | ACE^2^ 10 items | **(a)** Confidential environment **(b)** Staff Administered **(c)** Survey Administrators **(d)** No | **(a)** ✗ **(b)** Survey administrators were nurses instructed and supervised by a research field coordinator | **(1)** Used in analysis - Correlation/Regression analysis with health risk behaviors | ✗ | ✗ | ✗ |
| Nishida, 2015  (Dissertation) | 159/USA, Illinois | Cross-sectional study | **(a)** African American, %13.32; Asian American, 0.67%; Caucasian, 28.67%; Native American, 0.67% **(b)** North Latino American; 43.33%; Central Latino American, 6.67%; South Latino American, 6.67% **(c)** Female, 71.15%; Male, 28.85% | ACE^2^ 10 items | **(a)** Private room **(b)** Self-report (Staff assisted if needed) **(c)** Researcher **(d)** Yes | **(a)** Research assistants who spoke Spanish was trained in approaching patients and providing research materials in designated medical clinics **(b)** ✗ | **(2)** Used in analysis - Correlation/Regression analysis with disease diagnosis | If participants interested in learning about ACEs, resources, handouts, trusted website info, and resources for therapy sheet were shared. | Researchers were prepared to direct patients to therapist if they reported any distress. | ✗ |
| Njoroge et al., 2023 | 454/USA, New York | Cross-sectional study | **(a)** Hispanic, 54.2%; Black, 19.6%; Other, 26.2%  **(b)** -  **(c)** Female, 49.1%; Male, 36.6%; Non-binary, 14.3% | ACE^4^ 17 item | **(a)** Private room **(b)** Staff-Administered **(c)** Research team **(d)** Yes | **(a)** All research staff underwent training on interview techniques, including engagement, confidentiality, the research question, and the goals of the study.  **(b)** ✗ | **(1)** Used in analysis - Correlation/Regression analysis with health risk behaviors  **(2)** Used in analysis - Correlation/Regression analysis with disease diagnosis  **(3)** Identify healthcare access, nonattendance, literacy, referral for counseling services, protective factors, and/or social determinants of health  **(4)** Identify certain groups' ACEs patterns | ✗ | ✗ | ✗ |
| Peck et al., 2021 | 60/USA, Urban southern area | Quality improvement pilot study | **(a)** Black, 70%; White, 25% **(b)** Latino, 5% **(c)** Female, 30%; Male, 66.7%; Transgender Female, 3.3% | ACE^5^ 10 items | **(a)** Exam room **(b)** Staff administered **(c)** Nurse practitioner **(d)** No | **(a)** ✗ **(b)** ✗ | **(1)** Used in analysis - Correlation/Regression analysis with health risk behaviors | ✗ | ✗ | ✗ |
| Poole et al., 2017 | 3998/Canada, Alberta | Cross-sectional study | **(a)** Caucasian, 83%; African American, 1%; Asian, 9.9%; First Nations, 0.8%; Other, 5.2%  **(b)** - **(c)** Female, 68.2%; Male, 31.8% | ACE^2^ 10 items | **(a)** Mixed – on-site or off-site  **(b)** Self-report **(c)** NA **(d)** No | **(a)** ✗ **(b)** ✗ | **(2)** Used in analysis - Correlation/Regression analysis with disease diagnosis | ✗ | ✗ | ✗ |
| Priestley, 2023 (dissertation) | 71/USA, Vermont | Exploratory, descriptive study | **(a)** Caucasian, 100%  **(b)** - **(c)** Not specified | ACE^2^ 10 items | **(a)** Exam room **(b)** Self-report **(c)** NA **(d)** No | **(a)** ✗ **(b)** ✗ | **(2)** Used in analysis - Correlation/Regression analysis with disease diagnosis | ✗ | Availability of referrals to behavioral health related to ACEs screening related stress | ✗ |
| Purkey et al., 2018 | 26/Canada, Ontario | Qualitative Design | **(a)** Canada-born, 84.6%; Other or no answer, 15.4% **(b)** - **(c)** Female, 100% | ACE^2^ 10 items | **(a)** Off-site (telephone survey) **(b)** Staff Administered **(c)** Researcher **(d)** No | **(a)** ✗ **(b)** ✗ | **(2)** Used in analysis - Correlation/Regression analysis with disease diagnosis | ✗ | Participants were offered trauma and counseling resources. | ✗ |
| Pykare and Knox, 2022 | 192/USA, Ohio | Quality improvement study | **(a)** - **(b)** -  **(c)** Female, 49%; Male, 48.4%; Missing, 2.6% | ACE^2^ 10 items | **(a)** Waiting room **(b)** Self-report or staff administered for telehealth appointments **(c)** Intake nurse **(d)** No | **(a)** The training used an interactive webinar with explanations about ACEs and their importance, verified through an Outlook voting tool, and included a PowerPoint emailed to staff. **(b)** ✗ | **(5)** For medical provider to discuss ACEs score results during visit | ✗ | On-site BH referrals | Screening ACEs was an intervention. Researchers compared pre-ACE and post ACE BH referrals |
| Rose et el., 2016 | 12/USA, Maine | Pre- and post-intervention design | **(a)** White, 100% **(b)** **(c)** Female, 58.3%; Male, 41.7% | ACE^6^ 15 item | **(a)** Not mentioned (On-site) **(b)** Staff administered **(c)** Social worker **(d)** No | **(a)** ✗ **(b)** ✗ | **(3)** Identify healthcare access, nonattendance, literacy, referral for counseling services, protective factors, and/or social determinants of health | ✗ | Referrals to on-site social work | Focused on restoring self-management, enabling biopsychosocial change through counseling and multidisciplinary care plans in a safe, empowering environment. |
| Ross et al., 2020 | 226/Canada, Nova Scotia | Cross-sectional study | **(a)** White, 93.8%; First Nation, 2.2%; Black, 0.4%; Metis, 0.9%; Chinese, 0.9%; Latin American, 0.4%; Other; 1.3% **(b)** - **(c)** Female, 65%; Male, 33.2%; Did not disclose, 1.8% | ACE^2^ 10 items | **(a)** On site (not mentioned) **(b)** Self-report **(c)** NA **(d)** No | **(a)** ✗ **(b)** ✗ | **(1)** Used in analysis - Correlation/Regression analysis with health risk behaviors | ✗ | ✗ | ✗ |
| Scherrer et al., 2014 | 253/USA, Texas | Cross-sectional study | **(a)** White, 47.4%; Other, 52.6% **(b)** - **(c)** Female, 66%; Male, 34% | ACE^2^ 10 items | **(a)** Not mentioned (on-site) **(b)** Self-report **(c)** NA **(d)** Yes | **(a)** ✗ **(b)** ✗ | **(2)** Used in analysis - Correlation/Regression analysis with disease diagnosis | ✗ | ✗ | ✗ |
| Sinnott et al., 2015 | 2047/Ireland, Mitchelstown | Cross-sectional study | (a) - **(b)** -  **(c)** Female, 51%; Male, 49% | ACE^2^ 10 items | **(a)** Not mentioned ( On-site) **(b)** Self-report **(c)** NA **(d)** No | **(a)** ✗ **(b)** ✗ | **(2)** Used in analysis - Correlation/Regression analysis with disease diagnosis | ✗ | ✗ | ✗ |
| Sosnowski et al., 2023 | 531/USA, North Carolina and Florida | Prospective cohort study | **(a)** Black, 38%; white 62% **(b)** Hispanic/Latina, 22%; not Hispanic/Latina, 78% **(c)** Female, 100% | ACE^2^ 10 items | **(a)** Not mentioned (on-site) **(b)** Self-report **(c)** NA  **(d)** Yes | **(a)** ✗ **(b)** ✗ | **(1)** Used in analysis - Correlation/Regression analysis with health risk behaviors | ✗ | ✗ | ✗ |
| Strenth et al., 2022 | 581/USA | Cross-sectional study | **(a)** White, 49%; African American, 27.8%; Other, 18.3%; Asian, 3.2%; American Indian, 1.7% **(b)** Hispanic, 50.7%; Non-Hispanic, 49.3% **(c)** Female, 60.5%; Male, 39.5% | ACE^2^ 10 items | **(a)** Not mentioned (On-site) **(b)** Self-report **(c)** NA **(d)** No | **(a)** ✗ **(b)** ✗ | **(2)** Used in analysis - Correlation/Regression analysis with disease diagnosis | ✗ | ✗ | ✗ |
| Sukkarieh et al., 2023 | 300/Lebanon, Beirut | Cross-sectional study | **(a)** Not specified  **(b)** - **(c)** Female, 48%; Male, 52% | ACE^2^ 10 items | **(a)** Not mentioned (on-site) **(b)** Self-report **(c)** NA **(d)** No | **(a)** ✗ **(b)** ✗ | **(2)** Used in analysis - Correlation/Regression analysis with disease diagnosis  **(3)** Identify healthcare access, nonattendance, literacy, referral for counseling services, protective factors, and/or social determinants of health | ✗ | ✗ | ✗ |
| van den Berk-Clark et al., 2023 | 349/USA, Missouri | Cross-sectional study | **(a)** Black, 12.1%; White 79.5%; Other, 8.4% **(b)** Latino/Hispanic, 1.7%; Non-Hispanic/Latino, 98.3% **(c)** Female, 70.8%; Male, 28.5%; Other, 0.6% | ACE^2^ 17 items | **(a)** NA (off-site) **(b)** Self-report (Mail/ electronically) **(c)** NA **(d)** No | **(a)** ✗ **(b)** ✗ | **(2)** Used in analysis - Correlation/Regression analysis with disease diagnosis | ✗ | ✗ | ✗ |
| van Roessel et al., 2021 | 338/Canada, Alberta | Cross-sectional study | **(a)** First Nations, 0.6%; Arab/West Asian, 1.8%; Black, 9.2%; Asian, 16.9%; Latin American, 3.6%; South Asian, 10.7%; Southeast Asian, 1.5%; White 48.8%; Other, 0.6%; Missing, 6.5% **(b)** - **(c)** Female, 100% | ACE^2^ 10 items | **(a)** Not mentioned (On site) **(b)** Self-report **(c)** NA **(d)** No | **(a)** ✗ **(b)** ✗ | **(2)** Used in analysis - Correlation/Regression analysis with disease diagnosis | Patients received a pamphlet outlining health associations with ACEs and a follow-up discussion was encouraged by the healthcare professional | 18 women (5.3%) received a referral to a therapist or mental health clinician after ACEs screening | ✗ |
| Vishwanath and Maxwell, 2023 | 130/USA, California | Cross-sectional study | **(a)** White, 70%; Black, 17.7%; Asian, 3.8%; Pacific Islander, 1.5%; American Indian, 2.3%; Not listed, 4.6% **(b)** Not Hispanic, 66.2%; Hispanic, 30.8%; Declined, 3.1% **(c)** Female, 35.4%; Male, 64.6% | ACE^2^ 10 items | **(a)** Exam room  **(b)** Staff-administered **(c)** Medical Assistant  **(d)** No | **(a)** ✗ **(b)** ✗ | **(3)** Identify healthcare access, nonattendance, literacy, referral for counseling services, protective factors, and/or social determinants of health | Patients were given education and information for next steps to mitigate their risks of chronic health conditions. | Patients with four or more ACEs were referred by their provider to internal services such as psychiatry, behavioral health therapists, social workers, alcohol and drug counselors, and case managers. | If the ACEs score was four or more, providers provided ACEs-related counseling during the appointment. |
| Williams et al., 2018 | 210/USA | Cross-sectional study | **(a)** Black/ African American, 64%; White/European-American, 27%; Multiracial, 4%; Other, 2% **(b)** Latino/Hispanic, 2% **(c)** Female, 60%; Male, 40% | ACE^2^ 10 items | **(a)** Waiting room **(b)** Self-report **(c)** NA **(d)** No | **(a)** ✗ **(b)** ✗ | **(1)** Used in analysis - Correlation/Regression analysis with health risk behaviors | ✗ | ✗ | ✗ |
| Young-Wolff et al., 2019 | 584/USA, California | Cross-sectional study | **(a)** Non-Hispanic White, 63%; Non-Hispanic Black, 9.2%; Other, 4.1%; Unknown, 9.4% **(b)** Hispanic, 14.2% **(c)** Female, 3.2%; Male, 96.9% | ACE^2^ 10 items | **(a)** Not mentioned **(b)** Not mentioned **(c)** Not mentioned **(d)** No | **(a)** ✗ **(b)** ✗ | **(2)** Used in analysis - Correlation/Regression analysis with disease diagnosis | ✗ | ✗ | ✗ |
| Zak-Hunter et al., 2023 | 123/USA, Minnesota | Cross-sectional study | **(a)** Black/African American, 31.7%; Hispanic/Latino, 21.1%; Asian American, 23.5%; Native Hawaiian/Other Pacific Islander, 1.6%; American Indian/Native American, 10.6%; Other, 2.4%; Multiracial, 8.9% **(b)** - **(c)** Female, 100% | ACE^2^ 10 items | **(a)** Off-site^8^ **(b)** Self and staff administered^8^ **(c)** Research staff for surveys via phone^8^ **(d)** Yes^8^ | **(a)** ✗ **(b)** ✗ | **(1)** Used in analysis - Correlation/Regression analysis with health risk behaviors  **(2)** Used in analysis - Correlation/Regression analysis with disease diagnosis  **(3)** Identify healthcare access, nonattendance, literacy, referral for counseling services, protective factors, and/or social determinants of health  **(4)** Identify certain groups' ACEs patterns | ✗ | ✗ | ✗ |

*Abbreviations:* ACE, Adverse Childhood Experiences; PI, Primary Investigator; BH, Behavioral Health; PTSD, Post Traumatic Stress Disorder; SDH, Social Determinants of Health, NA, Not Applicable; TIC, Trauma-Informed Care.

^1^ WHO. (2020). Adverse Childhood Experiences International Questionnaire (ACE-IQ). World Health Organization. <https://www.who.int/publications/m/item/adverse-childhood-experiences-international-questionnaire-(ace-iq)#:~:text=The%20ACE%20International%20Questionnaire%20%28ACE-IQ%29%20is%20intended%20to.%20Accessed%2031%20Mar.%202023>.

^2^ Felitti, V. J., Anda, R. F., Nordenberg, D., Williamson, D. F., Spitz, A. M., Edwards, V., Koss, M. P., & Marks, J. S. (1998). Relationship of Childhood Abuse and Household Dysfunction to Many of the Leading Causes of Death in Adults. *American Journal of Preventive Medicine*, *14*(4), 245–258. <https://doi.org/10.1016/s0749-3797(98)00017-8>

^3^ Swingen, J. K. (2020). *Psychometric developments of the ACE-IQ: Understanding the trauma history of latine immigrants* [Dissertation]. <https://doi.org/10.25772/GV0T-VY89>

^4^ The survey included modified ACE questions on sexual abuse, verbal abuse, and mother treated violently to be more culturally sensitive, and added categories: parental separation/divorce, bullying, separation from parents, and living with foster parents.

^5^ Meinck, F., Cosma, A. P., Mikton, C., & Baban, A. (2017). Psychometric properties of the Adverse Childhood Experiences Abuse Short Form (ACE-ASF) among Romanian high school students. Child abuse & neglect, 72, 326–337. <https://doi.org/10.1016/j.chiabu.2017.08.016>

^6^ Rose, S. M., Hatzenbuehler, S., Gilbert, E., Bouchard, M. P., & McGill, D. (2016). A Population Health Approach to Clinical Social Work with Complex Patients in Primary Care. *Health & Social Work*, *41*(2), 93–100. <https://doi.org/10.1093/hsw/hlw013>

^7^ Patient demographics were not provided, although adult diabetes patients (n = 16) were screened. The study gathered consent forms from the providers (n = 4) for implementing the ACEs screening.

^8^ The study conducts a secondary analysis of data from the *Family Matters* study (Berge et al., 2017), where information related to data collection procedures was gathered.
